# A Time to Kill: Targeting Apoptosis in Cancer

**DOI:** 10.3390/ijms16022942

**Published:** 2015-01-28

**Authors:** Jean L. Koff, Sampath Ramachandiran, Leon Bernal-Mizrachi

**Affiliations:** Department of Hematology and Medical Oncology at the Winship Cancer Institute of Emory University, Atlanta, GA 30322, USA; E-Mails: jkoff@emory.edu (J.L.K.); sramac2@emory.edu (S.R.)

**Keywords:** targeting apoptosis, cancer

## Abstract

The process of apoptosis is essential for maintaining the physiologic balance between cell death and cell growth. This complex process is executed by two major pathways that participate in activating an executioner mechanism leading to chromatin disintegration and nuclear fragmentation. Dysregulation of these pathways often contributes to cancer development and resistance to cancer therapy. Here, we review the most recent discoveries in apoptosis regulation and possible mechanisms for resensitizing tumor cells to therapy.

## 1. Introduction

Apoptosis, an orchestrated event in which cells are programmed to die after receiving specific stimuli, is an important component of cell growth control [1]. Apoptosis is characterized by morphologic changes, such as chromatin condensation, nuclear fragmentation and reduction of cell volume (known as pyknosis) [2], as well as biochemical changes that include caspase activation, breakdown of DNA and protein and membrane surface modifications that allow the apoptotic cell to be recognized and engulfed by phagocytic cells [3]. When disrupted, an imbalance between life and death of cells can lead to cancer. Here, we review two tightly regulated pathways that converge to produce apoptosis, the mechanisms by which cancer cells evade it and the therapeutic efforts to target these mechanisms in order to induce apoptosis in cancer cells.

## 2. Pathways to Apoptosis

**Figure 1:**
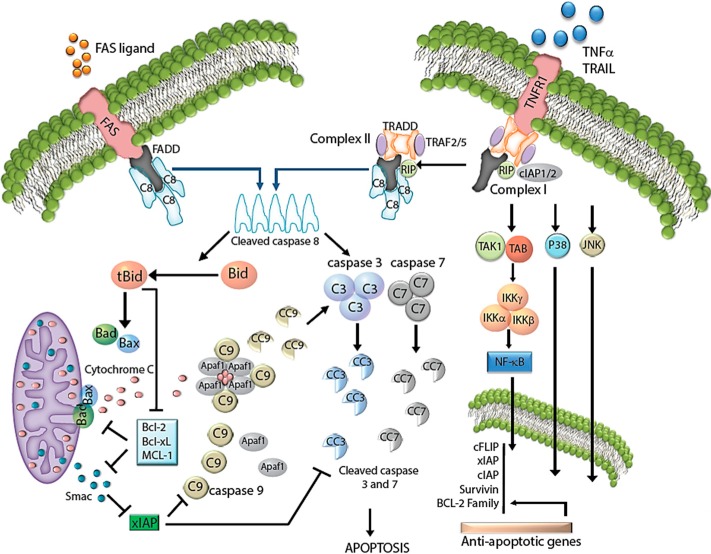
Apoptosis pathways in cancer. Activation of the death receptor pathway by FAS, TRAIL or TNFα leads to two mechanisms of downstream signaling. TRAIL or FAS ligand promotes the interaction of the FAS receptor with FADD and caspase-8 to form the DISC complex. TNFα promotes transient recruitment of the TNF receptor 1 (TNFR1) with TRADD, TRAF2, TRAF5, cIAP1/2 and RIP, which form signaling complex I. Complex I triggers a number of downstream signals, such as NF-κB, JNK and p38, to promote survival by inducing the expression of antiapoptotic genes, such as cFLIP, a common inhibitor of TNFR signaling. However, posttranslational modifications of complex I, such as RIP deubiquitination, can promote the dissociation of RIP1 and TRADD from the complex; they bind to FADD and caspase-8/-10 to form the apoptotic complex II. Formation of DISC or complex 2 elicits two mechanisms leading to cell death: direct induction of the executioner caspases-3 and -7 in type I cells and cleavage of Bid (tBID), which engages the intrinsic apoptotic pathway. In this pathway, stimuli, such as DNA damage or the presence of tBID, cause BAX and BAKk to interact and induce mitochondrial outer membrane permeability (MOMP). The resulting increase in MOMP leads to the release of cytochrome *c* and Smac into the cytoplasm. Cytochrome *c* recruits APAF1 and caspase-9 to promote caspase-9 auto-activation and subsequent activation of the executioner caspases. Smac interacts with xIAP to prevent the inhibition of caspase-9 and -3.

Apoptosis may be triggered by two major mechanisms: binding of death ligands to death receptors in the extrinsic pathway or cytotoxicity that initiates the intrinsic “mitochondrial” pathway [4]. Overall, these pathways converge to activate a series of cysteine aspartyl-specific proteases (caspases), which cleave key cellular proteins and dismantle the cell (Figure 1). Given the dire effects of caspase activation, it is necessary that these two pathways remain closely regulated at each step.

### 2.1. Extrinsic Pathway

The receptor-mediated (extrinsic) pathways transmit extracellular death signals to the apoptotic intracellular machinery to elicit cell death [1]. Death receptors comprise a subset of the tumor necrosis factor (TNF) receptor superfamily characterized by distinct protein motifs, namely death domains (DD) and death effector domains (DED). These specialized domains are capable of monovalent, homotypic interactions. On the cell surface, cognate ligands from the TNF family CD95 (first apoptotic signal, Fas/Apo1) and TNF-related apoptosis-inducing ligand (TRAIL) engage with one of the major death receptors to attract the DD-containing molecules, FADD (Fas-associated death domain protein) and/or TNF receptor-associated death domain (TRADD). Recruitment of FADD triggers pro-apoptotic pathways, while TRADD induces antiapoptotic signals. FADD attracts other DD/DED-containing proteins, such as pro-caspase-8 and -10, to promote the formation of the death-inducing complex (DISC) in the cytoplasmic compartment. In contrast, TRADD binds to and forms complex I with receptor interacting protein-1 (RIP1), TNF receptor-associated factor-2 (TRAF2), TRAF5 and the inhibitor of apoptosis protein-1 and -2 (cIAP1/2). This complex modulates pro-survival signals, such as those mediated by NF-κB, JNK and p38. However, in certain circumstances, RIP1 is deubiquitinated by the enzyme, cylindromatosis (CYLD), which leads to the dissociation of RIP1 and TRADD from complex I. RIP1 and TRADD then bind to FADD and caspases-8 and -10 to form complex II [5,6,7]. Once caspases-8 and -10 are activated, they relay and amplify the death signal, either through direct activation of the effector caspases-3, -6 and -7, commonly seen in lymphocytes (type I cells) or by promoting BID engagement of BAX and BAK to activate the intrinsic apoptotic pathway, a phenomenon commonly seen in type II cells, such as hepatocytes (Figure 1).

### 2.2. Regulation of the Extrinsic Pathway

The majority of signals that inhibit caspase-8 do so by affecting the recruitment of caspase-8 to the DISC complex. For example, cFLIP-long (cFLIPL) shares significant structural similarities with caspases-8 and -10, which allows it to compete for binding sites and thus displace caspases-8 and -10 from DISC. Similarly, A20 binding and inhibitor of NF-κB1 (ABIN1) exerts its antiapoptotic effect by affecting the interaction of RIP1 and FADD with caspase-8 [8].

Other mechanisms for the negative regulation of caspase-8 involve the induction of survival signaling pathways that later inhibit caspase-8 activation. cIAP1/2 contain a baculovirus IAP repeat (BIRD), a caspase-recruitment domain (CARD) and RING E3 ligases, which help to recruit TRAF1 and 2 and inhibit TNFα-apoptotic signaling. Although cIAP1/2 are inefficient caspase-8 inhibitors, they execute their inhibitory potential by inducing prosurvival signals, such as the NF-κB pathway. It is thought that this effect results from cIAP1/2 induction of RIPK1 ubiquitination and recruitment of TAK1, TAB2, TAB3 and the IKK complex [9].

In contrast, positive regulators of caspase-8 mediate their effect by inducing posttranslational modifications, such as ubiquitination. A clear example is the polyubiquitination of the p10 subunit of caspase-8 mediated by CUL3 upon induction by TRAIL. Polyubiquitinated caspase-8 recruits p62 to stabilize itself and increase its potency.

### 2.3. Intrinsic Pathway

Upon detection of cytotoxic internal stimuli, such as DNA damage or growth factor deprivation, two proapoptotic BCL2 proteins, BAX and BAK, undergo structural changes that lead to their activation. Both BAX and BAK migrate to the mitochondria, where they homodimerize to expose cryptic dimer-dimer binding sites and introduce pores into the surface of the mitochondria. This results in increased membrane permeabilization (MOMP) and the release of proteins from the mitochondrial intermembrane space (IMS) [10,11,12,13].

Of the many IMS proteins released during MOMP, cytochrome *c* is the most important, as it instigates apoptosome formation. To do so, cytoplasmic cytochrome *c* transiently binds the caspase adaptor molecule, Apaf-1, in the presence of ATP or dATP, which triggers oligomerization of Apaf-1 into a wheel-like heptamer that exposes its caspase activation and recruitment domains (CARDs) [14]. Consequently, Apaf-1 CARD domains bind to procaspase-9 CARDs to form the apoptosome. In this complex structure, procaspase-9 dimerizes and auto-activates. Activated caspase-9 then activates the executioner caspases-3 and -7 to perpetrate cell death within minutes (Figure 1, [15]).

### 2.4. Regulation of the Intrinsic Pathway

The intrinsic (or mitochondrial) pathway is tightly controlled by opposing actions of members of the BCL-2 family. These proteins, which each harbor at least one BCL-2 homology (BH) domain, are divided into three functionally-distinct groups: inhibitors of apoptosis (BCL-2, BCL-XL, BCL-W, MCL1, BCL-B and A1), which inhibit their pro-apoptotic counterparts; promoters of apoptosis (BAX, BAK and BOK); and regulatory BH3-only proteins (BAD, BIK, BID, HrK, BIM, BMF, NOXA and PUMA), whose conserved BH3 domain inhibits anti-apoptosis proteins and activates pro-apoptosis proteins [12,13,16]. Antiapoptotic BCL2 members inhibit intrinsic apoptotic signals by constraining the proapoptotic proteins, BAX and BAK, and, therefore, cytochrome *c* release. However, upon a cytotoxic stimulus, the effect of antiapoptotic BCL2 proteins is counteracted by BH3-only proteins, such as BIM or NOXA. BH3-only proteins release BAX-BAK from inhibition and allow them to promote MOMP and apoptosis (Figure 1).

The inhibitor of apoptosis (IAPs) protein family represents another negative regulator of the intrinsic apoptotic pathway. IAPs directly inhibit caspases by several mechanisms [17]. First, their BIR domains bind the active site of caspases’ active sites and inhibit proteolytic function, as do XIAP and survivin and cAP1/2 to caspases-3 and -7 [18]. Second, xIAP directly inhibits the activation of pro-caspase-9. Third, some IAPs may target effector caspases for ubiquitination and proteasomal degradation, thus limiting their executioner potential. In less direct mechanisms, cIAPs contribute to the activation of antiapoptotic signals, such as NF-κB and JUNK1. Thus, cIAP1/2 play a crucial role in regulating NF-κB activation during TNF signaling [19].

Other IMS proteins may facilitate caspase activation following MOMP by targeting IAP family members. Activation of the intrinsic apoptotic pathway induces the mitochondrial outer membrane to release Smac into the cytosol. There, Smac binds to various IAP proteins, mainly xIAP, and neutralizes their antiapoptotic effect by facilitating their degradation by proteasomes [20]. However, unlike cytochrome *c*, losing Smac, Omi (another proapoptotic IAP protein) or both proteins does not result in the inability to activate caspases or undergo apoptosis [21,22,23]. Given that other proteins have also been shown to inhibit xIAP, these findings indicate that there may be considerable redundancy in this regard (Figure 1, [24]).

More recently, the interplay between autophagy machinery and apoptosis has been described. Members of both systems share roles that serve as links between the two pathways. Regulatory members of the intrinsic pathway affect both MOMP and autophagy, whereas proapoptotic BCL2 proteins not only promote mitochondrial permeabilization, but also autophagy. In contrast, antiapoptotic BCL-2 proteins inhibit MOMP and autophagy. A clear example is the inhibition of autophagy that results from the binding of BCL-2/BCL-xL to Beclin-1, an important initiating factor of autophagy [25]. Reciprocally, members of the autophagy system can also affect the intrinsic apoptotic pathway. For instance, ATG5 enhances cell susceptibility to MOMP during treatment with DNA damaging agents. DNA damage induces calpain-mediated ATG5 cleavage, which results in a truncated ATG5 capable of binding to Bcl-xL and, thus, promoting cytochrome *c* release [26].

### 2.5. Lysosomal Mitochondrial Pathway

Lysosomal membrane permeabilization (LMP) is another process found to positively regulate apoptosis [27]. LMP results from different signals, such as death receptors, reactive oxygen species (ROS), ultraviolet radiation, proteasome inhibition, growth deprivation and p53 activation [28,29,30]. According to the initiating stimuli, death signals are transmitted to the lysosome in various forms, including through factors, such as BAX, BIM, BID and caspase-8, after death receptor activation, or in the case of p53 activation, through lysosome-associated apoptosis-inducing protein (LAPF) [27,30,31]. Upon partial or selective permeabilization, lysosomes release hydrolases, such as cathepsins, into the cytosol. These cathepsins may trigger caspase-dependent or -independent apoptosis contingent on the cell type, the context of the lethal signal, the amount of cathepsins released from the lysosome and the relative abundance of cathepsin inhibitors [27]. Relevant for this review, cathepsins can directly activate caspase-3 or facilitate MOMP. The link between LMP and MOMP seems to be mediated by the effect of cathepsin on BID and caspase-2. On the other hand, the caspase-independent mechanism seems to rely on the mitochondrial release of apoptosis-inducing factor (AIF). Once in the cytoplasm, AIF translocates to the nucleus, where it promotes chromatin condensation and DNA fragmentation [31]. In cancer cells, lysosomes demonstrate greater susceptibility to LMP [30]. This lower threshold for permeabilization is thought to result in part from the greater accumulation of iron-containing proteins and higher rates of ROS. Hence, the fact that cancer cells often evade apoptotic signals induced by the caspase cascade makes the lysosomal pathway an attractive opportunity for drug development in cancer.

## 3. Evasion of Apoptosis by Cancer Cells

Apoptosis is highly regulated in eukaryotic cells, since an inappropriate apoptosis trigger will endanger the cell’s survival. However, many cancer cells develop mechanisms to evade this tightly regulated cell death program by manipulating the levels of antiapoptotic molecules or inactivating proapoptotic cell death components [32]. The advent of genomic analysis has provided key insights into the various mutations that allow cancers to circumvent cell death. We now understand that cancer cells utilize a variety of mechanisms to escape apoptosis, some of which are specific to a particular tumor type and others that are employed by a number of different cancers. Dysregulation may occur in both the intrinsic and extrinsic pathways, examples of which are provided below.

### 3.1. Disruption of the First Apoptotic Signal (FAS) and DR5 Response

Several studies have demonstrated that anticancer therapy triggers the extrinsic pathway in an autocrine or paracrine manner via the FAS receptor. It is thus unsurprising that several malignancies, such as melanoma, esophageal cancer and pulmonary adenocarcinomas, downregulate the FAS receptor to evade apoptotic signaling. Even more remarkable are the presence of somatic mutations in the FAS receptor that allow tumors to evade the immune response. FAS receptor mutations have been described in tumors, such as nasal T/NK lymphomas (50%), testicular germ cell tumors (37.5%), non-small cell lung carcinoma (20.2%), melanoma (6.8%) and squamous cell carcinoma of the skin arising from burn scars (14%), among others [33].

Another mechanism that alters FAS-triggered apoptosis relies on amplification of decoy receptor 3, a protein that shares similar extracellular motifs to the TNF receptor and inhibits FAS-mediated apoptosis. Strikingly, half of all colon, lung and breast cancers exhibit amplification of this gene, suggesting that this mechanism may be essential for evading the immune response. Moreover, in some tumors, such as breast cancer, amplification of decoy receptor 3 is associated with inferior clinical outcomes [33].

Mutations in the TRAIL DR5 receptor were initially described in head and neck cancers. Additionally, such mutations have been reported in 10.6% of non-small cell lung cancer and 7% of gastric cancers. As the majority of these are missense mutations, most of them alter the death domain of DR5 or abrogate its capacity to activate death receptor signaling [33].

### 3.2. Disruption in the Balance of Bcl2 Proteins

Disturbance of the tightly regulated balance between Bcl2 family members, either by decreased expression of pro-apoptotic proteins, overexpression of anti-apoptotic proteins or both, can prevent caspase-9 activation. For instance, most follicular lymphomas are characterized by the translocation t(14,18), which places the *bcl2* gene next to the promoter region of the immunoglobulin heavy chain gene, resulting in constitutive expression of anti-apoptotic Bcl2 [34]. This translocation is also seen in other hematologic malignancies, including chronic lymphocytic leukemia (CLL) and diffuse large B-cell lymphoma [35,36]. In other lymphomas, the *Bcl2* gene may be amplified or transcriptionally upregulated by mechanisms that remain unclear. Interestingly, t(14,18) *per se* has been shown to be insufficient for malignant transformation, as many healthy individuals have circulating memory B-cells harboring that translocation [37].

On the other hand, inactivation of pro-apoptotic Bcl2 proteins may also contribute to the escape of cancer cells from death. One of the most frequently mutated genes in microsatellite-unstable colon cancer is BAX, which is inactivated via frameshift mutation in ~50% of such cancers [38]. Similarly, missense mutations in BAD have been described in sporadic colon cancer, albeit at a much lower rate [39].

### 3.3. Dysregulation of Inhibitor of Apoptosis (IAPs)

Many cancers exhibit aberrancies in the expression of IAPs. Perhaps the most striking example of IAP overexpression in cancers is that of survivin, an IAP limited to embryonic tissues and not normally found in adult differentiated cells, but described in a variety of tumor types [40]. *In vitro* work demonstrates that cells overexpressing survivin are less susceptible to apoptosis, and patient studies confirm that overexpression of survivin in tumor samples correlates with worse prognosis and higher rates of treatment failure and relapse [41]. Amplification of the IAP gene loci *cIAP1* and *cIAP2* is also seen in many carcinomas, as well as glioblastomas [17]. Mouse studies of these genes provide evidence for their role as proto-oncogenes. Conversely, downregulation of the ubiquitously expressed XAF1, a negative regulator of XIAP, is described in several cancer lines, and promoter methylation has been shown to cause XAF1 silencing in some gastric cancers [42].

Because certain IAPs regulate other processes, such as cytokinesis and signal transduction, dysregulation of these genes sometimes circumvents apoptosis through signaling pathways rather than through the direct inhibition or ubiquitination of caspases by IAPs. For instance, *in vitro* work suggests that survivin may promote oncogenesis through a non-apoptotic pathway by disrupting cellular mitotic spindle checkpoints [43]. Furthermore, deletion (not amplification) of *cIAP1* and *cIAP2* in multiple myeloma results in activation of the noncanonical NF-κB pathway, which, in turn, results in upregulated expression of anti-apoptotic proteins, such as BCL-XL, BCL2, XIAP and A1 [44,45,46].

### 3.4. Reduced Caspase Activity

Initiator and effector caspases are essential for the execution of apoptosis, making them prime subjects in elucidating how cancer cells evade apoptosis. Several epidemiologic studies have investigated correlations between caspase gene polymorphisms and cancer risk, but how particular polymorphisms contribute to caspase function and expression has yet to be determined in many cases [33]. Large population studies investigating single nucleotide polymorphisms (SNP) in caspases-9 and -3 (rs4647601) demonstrated some predisposition towards such SNPs in lung cancer or head and neck carcinomas [33].

Although mutations in all caspases have been found in several solid tumors, their roles in tumor initiation or resistance to treatment are not well defined. Notably, sequencing analysis across 21 different tumor kinds demonstrated that caspase-8 was significantly altered in colon, gastric and head and neck cancers [47]. A small proportion of gastric carcinomas have been found to carry mutations in caspases-1, -5 and -8. Similarly, mutations in caspase-3 and -7 have been identified in tumors of the head and neck, in caspases-3, -4, -5 and -7 in colon cancer and in caspases-3 and -5 in lung cancer [33].

## 4. Targeting Apoptosis Pathways in Cancer Treatment

### 4.1. TNF-Related Apoptosis-Inducing Ligand (TRAIL)

Since TRAIL has been shown to induce cell death in cancer cells compared to normal cells, TRAIL receptor and TRAIL ligands are attractive targets for anti-cancer therapy. Additionally, recombinant TRAIL ligand and antibodies against the TRAIL receptor have been shown to be safe at very high doses [48]. Unfortunately, several clinical trials using such drugs as single agents to induce cancer cell death have failed to reproduce the promising results obtained in animal models. This has led to several preclinical studies investigating TRAIL-based multimodality therapy to maximize antitumor activity. Both conventional chemotherapy with DNA damaging agents and radiotherapy have been shown to induce TRAIL receptors, thus suggesting the possibility of a synergistic effect when such therapies are combined with TRAIL-targeted treatment [48,49]. However, there is a major caveat in the use of TRAIL-based therapy for cancer treatment: given the presence of both agonistic receptors (TRAIL-R1 and TRAIL-R2) and antagonistic decoy receptors (TRAIL-R3 and TRAIL-R4) in human cells, recombinant TRAIL ligand may be either proapoptotic or antiapoptotic. Moreover, in some cancer cells, TRAIL actually induces NF-κB, thus promoting cancer cell survival rather than apoptosis [48]. Thus, the context-based effect of TRAIL signaling may explain the lack of efficacy seen in recent studies of TRAIL-targeted anticancer therapy.

### 4.2. Bcl2 Family

Given the crucial role of Bcl2 family members in regulating apoptosis, a substantial effort has been devoted to developing drugs that mimic their common BH3 domain, with the goal of inducing apoptosis by preventing inhibitory binding to pro-apoptotic BAX and BAK by their pro-survival counterparts. ABT-737 and its oral derivative, ABT-263, now called navitoclax, were among the first promising BH3 mimetics in cancer therapy [50,51]. Both drugs avidly bind and inhibit BCL-2, BCL-XL and BCL-W, but not MCL-1 or A1. Interestingly, their chief target in cells with high levels of BCL-2 was found to be BIM-BCL-2 complexes rather than unoccupied BCL-2, with better inhibition of BIM-BCL-2 complexes than BIM-BCL-XL or BIM-BCL-W complexes [52]. Initial clinical trials of single-agent navitoclax have demonstrated significant activity in B-cell malignancies, especially CLL [53], and several preclinical studies evaluating the addition of navitoclax to conventional cytotoxic agents or targeted therapy show promising results in both solid tumor and hematologic malignancy models [54,55]. However, the practical use of navitoclax has been limited due to its propensity to induce acute thrombocytopenia. ABT-199, a newer BH3 mimetic that specifically targets BCL2, shows similar activity in lymphoid malignancies without the adverse effect of thrombocytopenia [56,57]. Preclinical studies in breast cancer suggest that ABT-199 enhances the killing effect of conventional chemotherapy [58] and posit a role for ABT-199 in the treatment of multiple myeloma cases harboring the (11,14) translocation [59]. The most promising advances using ABT-199 have occurred in the treatment of CLL [60], in which ABT-199 alone or in combination with the anti-CD20 monoclonal antibody, rituximab, demonstrated an impressive overall response rate in relapsed/refractory CLL patients with high risk features. However, this success has been accompanied by evidence of tumor lysis syndrome, a considerable side effect associated with cell death in the setting of a large tumor burden [56]. In spite of this, clinical trials continue, and phase III trials are planned. Other effective BH3 mimetics targeting MCL-1 and A1 have been less successful, but research remains ongoing [61].

### 4.3. IAP Inhibitors

The potential capacity of Smac to promote cell death led to the development of several small molecule mimetics of Smac in pursuit of a novel anticancer therapeutic approach [62]. Early studies using the Smac mimetic, LBW242, explored its use in multiple myeloma models *in vitro* and in xenograft mouse models [63]. Their findings suggested that LBW242 synergizes with other compounds, such as bortezomib, TRAIL or DNA damaging agents, such as melphalan, to reduce tumor burden. Along these lines, LBW242 was also highly beneficial in a FLT3-mutated acute myelogenous leukemia (AML) xenograft mouse model when administered along with the protein kinase inhibitor, PKC412 [64]. Recent studies also showed the efficacy of the Smac mimetic, BV6, in combination with glucocorticoids (GC) in childhood acute lymphocytic leukemia (ALL). Similarly, combination of BV6 with demethylating agents 5-azacytidine or 5-aza-2'-deoxycytidine induced cell death by a novel necroptosis mechanism in AML models resistant to apoptosis [65,66]. Another Smac mimetic compound, SM-164, has improved the efficacy of radiosensitization in head and neck squamous cell carcinoma [67] and showed effectiveness in combination with doxorubicin in hepatocellular carcinoma cells [68]. Recently, the novel Smac mimetic, RMT5265.2HCL (RMT), which binds to XIAP with similar affinity to that of endogenous Smac, has been shown to induce apoptosis in virally-associated lymphoma models that are otherwise resistant to receptor-mediated apoptosis [69]. Investigation of this drug’s mechanism showed that in addition to inhibiting IAP protein function, RMT also induces the release of endogenous Smac and cytochrome *c* from mitochondria, which could enhance its efficacy. A new, highly potent Smac mimetic, known as SM-1200, was reported to inhibit cell growth *in vitro* and in MDA-MB-231 breast cancer xenograft models, laying the groundwork for clinical trials of single-agent Smac mimetics in the near future [70]. Taken together, there is much interest in pursuing Smac mimetics as a possible addition to our anti-cancer armamentarium.

## 5. Conclusions

As research further defines the intricate nuances underlying apoptotic pathways and the ways in which they are dysregulated in cancer, our ability to design effective therapies targeting such mechanisms grows. Although dysregulated apoptosis in malignant cells remains an attractive target in cancer therapy, much work needs to be done to realize the full potential of such approaches. Most therapeutics remain in the preclinical stages, and several recent clinical trials have been plagued either by dose-limiting effects or by disappointing responses. However, promising results maintain excitement about these approaches, and further efforts should focus on maximizing potency, while minimizing side effects.
